# Maternal and Fetal Outcomes of Contemporary Obstetric Interventions: A Systematic Review

**DOI:** 10.7759/cureus.114514

**Published:** 2026-08-13

**Authors:** Nikeeta Ashokrao Khanorkar, Sayantan Choudhury, Mimansa Bharti, Sujit Das, Jewel Jacob, Krishna Meher, Bhaskar Jyoti Paul

**Affiliations:** 1 Department of Obstetrics and Gynaecology, Government Medical College, Nagpur, IND; 2 Department of Obstetrics and Gynaecology, Agartala Government Medical College (AGMC), Agartala, IND; 3 Department of Obstetrics and Gynaecology, Maharani Laxmibai Medical College, Jhansi, IND; 4 Department of Obstetrics and Gynaecology, Agartala Government Medical College, Agartala, IND; 5 Department of Obstetrics and Gynaecology, TSC Hospital, Thiruvananthapuram, IND; 6 Department of Prasuti and Stree Roga, Sri Sri Nrusinghnath Ayurved College and Research Institute, Bargarh, IND; 7 Department of Obstetrics and Gynaecology, College of Medicine, University of Shaqra, Shaqraa, SAU

**Keywords:** cesarean delivery, fetal monitoring, labour induction, neonatal outcomes, obstetric interventions

## Abstract

Labour and delivery interventions play an important role in maternal and neonatal care, particularly when labour progress is delayed, fetal well-being is uncertain, or high-risk obstetric conditions are present. Current literature often examines these interventions separately, whereas clinical practice typically involves overlapping decisions regarding induction, augmentation, monitoring, caesarean timing, operative delivery, and newborn care. This review narratively summarised findings from 10 completed studies examining maternal, delivery, surgical, and neonatal outcomes across distinct obstetric interventions. As each intervention was represented by only one or two primary studies, the findings were presented as intervention-specific evidence and interpreted with appropriate caution. A systematic review approach was followed, using the Preferred Reporting Items for Systematic Reviews and Meta-Analyses (PRISMA) guidelines. Database searching identified 337 records; after duplicate removal, screening, and eligibility assessment, 10 studies were included. The studies covered labour induction, oxytocin augmentation, electronic fetal monitoring, placenta previa, placenta accreta spectrum, caesarean delay, operative vaginal delivery, episiotomy, manual rotation, and essential newborn care practices. Combined misoprostol and Foley balloon use shortened the induction-to-delivery time, while sequential Foley use after misoprostol was linked to longer labour and a higher rate of caesarean delivery. Continuous fetal monitoring increased operative delivery without a clear neonatal benefit in low-risk labour. Planned delivery improved outcomes in placenta previa and placenta accreta spectrum, while caesarean delay increased severe maternal outcomes. Mediolateral episiotomy reduced obstetric anal sphincter injury in selected operative deliveries. Overall, timely, selective, and risk-based obstetric care appears essential for improving maternal and neonatal safety.

## Introduction and background

Clinical interventions during labour and delivery are used in response to specific maternal, fetal, or labour-related indications. Among women undergoing labour induction, concurrent or sequential administration of vaginal misoprostol and a Foley catheter has been evaluated for its effects on cervical ripening, induction duration, and mode of delivery [1,2]. Oxytocin augmentation has been studied using different dosing regimens and in relation to labour progression and delivery outcomes [3,4]. In emergency caesarean delivery, the decision-to-delivery interval has also been examined as a determinant of maternal and fetal outcomes [5]. These intervention-specific studies indicate that obstetric outcomes may vary according to the clinical indication, intervention protocol, timing of care, and characteristics of the population studied. Evidence from individual studies should therefore be interpreted within the specific clinical context in which each intervention was evaluated [5]. Induction and augmentation methods may produce different outcomes according to their sequence, dosage, and clinical indication [6]. Combined pharmacological and mechanical cervical ripening has been evaluated for reducing induction duration, whereas sequential Foley catheter use after misoprostol has been associated with longer labour, caesarean delivery, and neonatal intensive care unit admission in selected populations [6]. Oxytocin augmentation may support labour progression when uterine contractions are inadequate, although its effects depend on dosage, timing, maternal condition, fetal status, and baseline labour characteristics [7]. Continuous electronic fetal monitoring is intended to facilitate the detection of fetal compromise during labour; compared with intermittent auscultation, it may increase caesarean and instrumental vaginal delivery rates without reducing overall perinatal mortality, although neonatal seizures may be reduced [6].

The timing and organisation of delivery are particularly relevant in high-risk obstetric conditions, including placenta previa, placenta accreta spectrum, delayed caesarean delivery, and emergency presentation [3]. Planned delivery in placenta previa and placenta accreta spectrum permits advance preparation for anaesthesia, blood product availability, neonatal support, and potential major surgery [3,4]. Emergency presentation, delayed intervention, and late recognition of placenta accreta spectrum have been associated with maternal and neonatal complications, although these associations may partly reflect the greater clinical severity of women requiring urgent delivery [4,5]. In resource-limited settings, consent processes, financial constraints, referral delays, operating-room availability, and provider-related factors may further contribute to delayed caesarean delivery [5]. These health-system barriers may influence maternal and neonatal outcomes independently of the intervention itself [8].

Operative vaginal delivery may facilitate expedited birth during the second stage of labour but may also be associated with maternal perineal trauma and neonatal complications [9]. Mediolateral episiotomy has been evaluated as a selective measure for reducing obstetric anal sphincter injury during operative vaginal delivery and should not be interpreted as supporting routine use [9]. Manual rotation of an occiput posterior fetal position is intended to facilitate vaginal birth, although randomised evidence has not consistently demonstrated an increase in spontaneous vaginal delivery or a reduction in operative birth [10]. These findings illustrate the importance of evaluating obstetric procedures according to demonstrated clinical outcomes rather than theoretical benefit alone [11].

Immediate newborn care practices also influence early neonatal outcomes, particularly in settings with high neonatal mortality [8]. Clean delivery practices, early breastfeeding, skin-to-skin contact, thermal protection, and skilled newborn assessment are important components of care around birth [8]. Outcomes remain influenced by gestational age, birth weight, fetal compromise, maternal complications, place of delivery, and severity of neonatal illness [12]. Current evidence supports the timely, selective, and risk-based use of labour and delivery interventions rather than uniform application across all patients [13]. Planned care may be advantageous in selected high-risk pregnancies, whereas unnecessary escalation in low-risk labour may increase operative delivery without consistent neonatal benefit [14].

Previous studies have generally evaluated induction, augmentation, monitoring, operative delivery, caesarean timing, and newborn care as separate clinical topics, although these decisions frequently overlap in practice [15]. The knowledge gap is therefore not the absence of intervention-specific studies but the limited synthesis of findings across the wider continuum of labour, delivery, and immediate newborn care. This review brings together these distinct areas to identify recurring principles concerning patient selection, timing, monitoring, escalation of care, and avoidance of unnecessary intervention. Given the heterogeneity of the evidence, the review does not seek to directly compare unrelated interventions or estimate a common treatment effect.

Objective of the review

This review aimed to narratively summarise evidence published between 2020 and 2025 on maternal, delivery, surgical, fetal, and neonatal outcomes associated with contemporary labour and delivery interventions. The review examined pregnant women in labour, women undergoing caesarean or operative vaginal delivery, and neonates receiving immediate birth care. The interventions and exposures included labour induction, cervical ripening, oxytocin augmentation, fetal monitoring, caesarean timing and delay, placenta-related delivery management, operative vaginal procedures, and immediate newborn care practices. Comparators were defined within the individual studies, and the outcomes included maternal morbidity, delivery mode, surgical complications, fetal condition, and early neonatal outcomes. The purpose was to identify intervention-specific findings and broader principles relevant to safe, timely, individualised, and context-sensitive obstetric care rather than to produce a pooled estimate across clinically dissimilar interventions.

## Review

Methodology

Study Design

The study was a systematic review of the published literature that included various labour, delivery, and early neonatal outcomes. The review was conducted following a standardised process for study identification, screening, eligibility assessment, and inclusion, in accordance with the Preferred Reporting Items for Systematic Reviews and Meta-Analyses (PRISMA) framework (PRISMA 2020 flow diagram) [16]. Studies with randomised, prospective, retrospective, registry-based, database-based, and propensity score designs were included in the review. There was variation in the population, intervention, comparator, and outcome measures across the 10 studies included, so a narrative synthesis was used rather than a meta-analysis. The latter enabled appropriate comparison of the various obstetric and neonatal studies with clinical diversity. No prospective protocol was registered for this review.

Search Strategy

A systematic literature search was conducted in PubMed/MEDLINE, Scopus, and Web of Science for studies published from January 1, 2020, to December 31, 2025. The final search was completed on June 10, 2026. Google Scholar was used as a supplementary source to identify additional potentially relevant studies. The search terms covered labour induction, cervical ripening, misoprostol, Foley balloon, oxytocin augmentation, electronic fetal monitoring, intermittent auscultation, caesarean delivery and delay, decision-to-delivery interval, placenta previa, placenta accreta spectrum, operative vaginal delivery, episiotomy, manual rotation, newborn care, and maternal, fetal, and neonatal outcomes. Terms were combined using the Boolean operators “AND” and “OR” and adapted to the syntax and field requirements of each database. PubMed/MEDLINE yielded 112 records, Scopus yielded 86 records, Web of Science yielded 74 records, and Google Scholar contributed 65 potentially relevant records, resulting in 337 records overall. The Google Scholar count represented records retained after supplementary relevance screening rather than the total number of results displayed by the platform. After 39 duplicate records were removed, 298 records underwent title and abstract screening. Sixty-three full-text articles were assessed for eligibility, of which 53 were excluded. Ten studies satisfied the predefined eligibility criteria and were included in the narrative synthesis. Complete database-specific search strings, limits, and the Google Scholar screening procedure are presented in Table 1. 

**Table 1:** Database-specific search strategies and limits

Database	Search strategy	Limits and screening procedure	Final search date
PubMed/MEDLINE	(("labour induction"[Title/Abstract] OR "labor induction"[Title/Abstract] OR "cervical ripening"[Title/Abstract] OR misoprostol[Title/Abstract] OR "Foley balloon"[Title/Abstract] OR "oxytocin augmentation"[Title/Abstract] OR "electronic fetal monitoring"[Title/Abstract] OR "intermittent auscultation"[Title/Abstract] OR "cesarean delivery"[Title/Abstract] OR "caesarean delivery"[Title/Abstract] OR "cesarean delay"[Title/Abstract] OR "decision-to-delivery interval"[Title/Abstract] OR "placenta previa"[Title/Abstract] OR "placenta accreta spectrum"[Title/Abstract] OR "operative vaginal delivery"[Title/Abstract] OR episiotomy[Title/Abstract] OR "manual rotation"[Title/Abstract] OR "newborn care"[Title/Abstract]) AND ("maternal outcome"[Title/Abstract] OR "maternal morbidity"[Title/Abstract] OR "fetal outcome"[Title/Abstract] OR "neonatal outcome"[Title/Abstract] OR "neonatal morbidity"[Title/Abstract] OR "early neonatal mortality"[Title/Abstract]))	English language; publication dates from January 1, 2020, to December 31, 2025	June 10, 2026
Scopus	TITLE-ABS-KEY(("labour induction" OR "labor induction" OR "cervical ripening" OR misoprostol OR "Foley balloon" OR "oxytocin augmentation" OR "electronic fetal monitoring" OR "intermittent auscultation" OR "cesarean delivery" OR "caesarean delivery" OR "cesarean delay" OR "decision-to-delivery interval" OR "placenta previa" OR "placenta accreta spectrum" OR "operative vaginal delivery" OR episiotomy OR "manual rotation" OR "newborn care") AND ("maternal outcome" OR "maternal morbidity" OR "fetal outcome" OR "neonatal outcome" OR "neonatal morbidity" OR "early neonatal mortality"))	English language; publication years 2020-2025	June 10, 2026
Web of Science	TS=(("labour induction" OR "labor induction" OR "cervical ripening" OR misoprostol OR "Foley balloon" OR "oxytocin augmentation" OR "electronic fetal monitoring" OR "intermittent auscultation" OR "cesarean delivery" OR "caesarean delivery" OR "cesarean delay" OR "decision-to-delivery interval" OR "placenta previa" OR "placenta accreta spectrum" OR "operative vaginal delivery" OR episiotomy OR "manual rotation" OR "newborn care") AND ("maternal outcome" OR "maternal morbidity" OR "fetal outcome" OR "neonatal outcome" OR "neonatal morbidity" OR "early neonatal mortality"))	English language; publication years 2020-2025	June 10, 2026
Google Scholar	Focused combinations of the principal intervention and outcome terms were used, including “labour induction maternal neonatal outcomes,” “electronic fetal monitoring delivery neonatal outcomes,” “placenta accreta planned emergency delivery,” “cesarean delay maternal neonatal outcomes,” “operative vaginal delivery episiotomy outcomes,” “manual rotation delivery outcomes,” and “newborn care early neonatal mortality.”	Publication years 2020-2025; the first 100 results ranked by relevance for each focused query were screened, and 65 potentially relevant records were retained	June 10, 2026

Inclusion Criteria

Studies were included if they were published between January 2020 and December 2025 and directly evaluated labour, delivery, operative, surgical, maternal, fetal, or immediate neonatal outcomes related to contemporary obstetric interventions. Eligible populations included pregnant women of reproductive age, women in labour, women undergoing caesarean delivery or operative vaginal delivery, and neonates whose outcomes were directly linked to labour, delivery, or immediate newborn care practices. Studies involving adult pregnant women, generally aged 18 years or older, were considered eligible. The eligible interventions or exposures included labour induction, cervical ripening, oxytocin augmentation, fetal monitoring, caesarean delivery timing, caesarean delay, placenta previa, placenta accreta spectrum, operative vaginal delivery, episiotomy, manual rotation, and early newborn care practices. Randomised trials, prospective cohort studies, retrospective cohort studies, case-control studies, registry-based studies, database-based studies, and propensity score analyses were included. Only English-language full-text studies with extractable maternal, fetal, neonatal, delivery, surgical, or procedural outcome data were included in the final evidence synthesis.

Exclusion Criteria

Studies were excluded if they were published before 2020, were not available in English, did not provide full-text access, or did not report extractable maternal, fetal, neonatal, delivery, surgical, or procedural outcomes. Reviews, systematic reviews, meta-analyses, editorials, commentaries, letters, conference abstracts, protocols, case reports, and case series were excluded from the main evidence synthesis. Review articles, systematic reviews, and meta-analyses were used only for background framing, contextual interpretation, and discussion support. Studies were also excluded if they did not focus directly on labour, delivery, obstetric interventions, caesarean delivery, operative vaginal delivery, placenta-related delivery complications, or immediate newborn care practices. Studies involving non-pregnant populations, gynaecological conditions unrelated to delivery care, antenatal screening without delivery outcomes, postpartum outcomes not linked to labour or delivery management, or neonatal outcomes not linked to obstetric or immediate newborn care practices were excluded. Duplicate records were removed before screening. At the full-text stage, 53 studies were excluded: 33 did not meet the inclusion criteria, 10 had insufficient outcome data, and 10 were not published in English.

Study Selection

All identified records were checked for duplication before screening. The remaining records were screened by title and abstract against the predefined eligibility criteria, followed by full-text assessment of potentially eligible articles. Eligibility decisions and uncertain cases were reviewed by the author team and resolved through discussion and consensus. No automated screening tools were used. Final inclusion was restricted to recent full-text original studies that directly matched the review objective, used an eligible analytical design, and provided extractable maternal, fetal, delivery, surgical, operative, or immediate neonatal outcome data. Ten studies met the final eligibility criteria and were included in the narrative synthesis. The complete selection process is presented in the PRISMA flow diagram.

Data Extraction

A structured data-extraction form was used for each included study. Extracted information included author, publication year, country or setting, study design, sample size, study population, intervention or exposure, comparator, outcomes assessed, and key findings. Maternal and delivery-related outcomes included caesarean delivery, operative delivery, severe maternal outcomes, hysterectomy, maternal morbidity, and obstetric anal sphincter injury. Neonatal outcomes included neonatal intensive care unit admission, Apgar score, neonatal morbidity, neonatal death, poor condition at birth, invasive ventilation, and early neonatal mortality. The extracted data were reviewed by the authors for completeness and consistency. Any discrepancies identified during verification were resolved through discussion and reference to the original full-text article. Eligibility assessment and data extraction were based on the published full-text reports; study authors were not contacted for additional or missing information. Extracted data were organised into tables to facilitate study-specific comparison.

Risk of Bias Assessment

Risk of bias was assessed using design-specific tools. The randomised controlled trial was assessed using the Cochrane Risk of Bias 2 (RoB 2) tool across five domains: randomisation process, deviations from intended interventions, missing outcome data, outcome measurement, and selection of the reported result [17]. Judgements were classified as low risk, some concerns, or high risk. The nine non-randomised studies were evaluated using the Risk of Bias in Non-randomised Studies of Interventions (ROBINS-I) tool across seven domains: confounding, participant selection, intervention classification, deviations from intended interventions, missing data, outcome measurement, and selection of the reported result [18]. Domain-level judgements were classified as low, moderate, serious, or critical risk of bias.

Data Synthesis

Due to substantial clinical and methodological heterogeneity across the included studies, a narrative synthesis was undertaken. Differences in populations, interventions, comparators, outcome definitions, and study designs precluded pooled statistical analysis and valid cross-study comparisons of raw percentages. Findings were organised into two broad categories: labour induction, augmentation, and fetal monitoring; and planned delivery, emergency care, and operative procedures. Quantitative findings were interpreted only within each study’s intervention-versus-comparator framework. Studies were compared according to intervention category, direction of association, reported outcome differences, clinical relevance, and risk of bias. No common effect estimate or statistical heterogeneity measure was calculated because the interventions and outcomes were not sufficiently comparable.

Results

Study Selection

**Figure 1:**
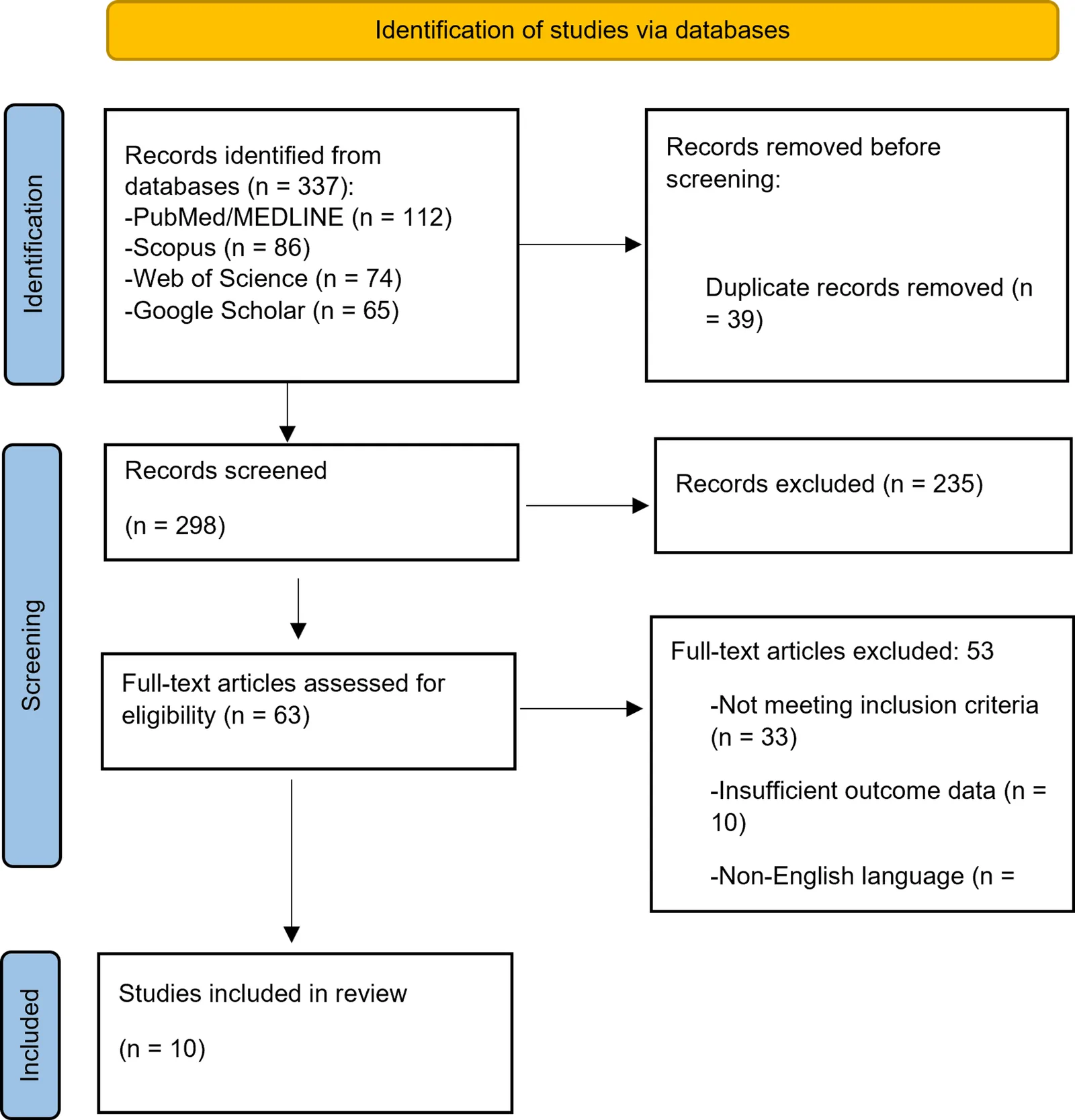
PRISMA flow diagram of study selection PRISMA: Preferred Reporting Items for Systematic Reviews and Meta-Analyses.

A total of 337 records were identified through database searches. Thirty-nine duplicate records were removed before screening, resulting in 298 records being screened by title and abstract. Of these, 235 records were excluded because they were not relevant to the review question and/or did not meet all the initial screening criteria. Sixty-three full-text articles were assessed for eligibility. The full-text review excluded 53 studies: 33 did not meet the inclusion criteria, 10 lacked adequate outcome data, and 10 were non-English studies. Ten studies met the inclusion criteria and were reviewed. The entire selection pathway is outlined in the PRISMA flow diagram (Figure 1).

Study Characteristics

The 10 studies included were published between 2020 and 2025 and conducted across a variety of obstetric and neonatal care settings. The studies were conducted in China, Ethiopia, Turkey, India, Somaliland, the USA, Poland, France, and multicountry (Global Network) sites. Study designs consisted of prospective cohort, retrospective cohort, registry-based cohort, case-control/database, propensity-score observational, and randomised controlled trial designs. The interventions studied were labour induction, augmentation of labour, fetal monitoring, timing of caesarean section, operative delivery, and neonatal care. The study features are summarised in Table 2. 

**Table 2:** Main characteristics of included studies BMI: body mass index; EFM: electronic fetal monitoring; h: hours; ICU: intensive care unit; NICU: neonatal intensive care unit; OASIs: obstetric anal sphincter injuries; PAS: placenta accreta spectrum.

Study	Country/setting	Study design	Sample/population	Exposure/intervention	Comparator	Main outcomes	Key findings
Zheng et al. [19]	China	Prospective cohort	199-term singleton pregnancies undergoing induction	Vaginal misoprostol plus Foley balloon	Vaginal misoprostol alone	Induction-to-delivery interval, delivery mode, satisfaction, neonatal outcomes	Combined misoprostol and Foley balloon shortened induction-to-delivery time and increased vaginal delivery within 24 h, but caused more induction-related pain.
Kebede et al. [20]	Ethiopia	Prospective follow-up study	588 low-risk labouring mothers	Continuous electronic fetal monitoring	Intermittent auscultation with Pinard fetoscope	Mode of delivery and immediate neonatal outcomes	Continuous EFM increased cesarean and instrumental delivery due to non-reassuring fetal heart rate patterns, without significant improvement in immediate neonatal outcomes.
Sertel et al. [21]	Turkey	Retrospective cohort	128 pregnancies complicated by placenta previa, including PAS and non-PAS subgroups	Emergency cesarean delivery	Planned cesarean delivery	Obstetric, neonatal, and surgical outcomes	Emergency cesarean delivery was associated with worse neonatal outcomes and more hysterectomies, especially among PAS cases.
Birendra and Jigyasa [22]	India	Retrospective cohort	32 women with placenta accreta spectrum	Planned or antenatally detected PAS management	Emergency or intraoperatively detected PAS	Maternal morbidity, hysterectomy, transfusion, ICU admission, neonatal outcomes	PAS was commonly associated with previous cesarean section and placenta previa. Planned/antenatally detected cases had better neonatal outcomes than emergency cases.
Kiruja et al. [23]	Somaliland	Prospective cohort	1,255 women undergoing cesarean section	Cesarean delay of 1–3 h or >3 h	No delay, defined as <1 h	Severe maternal outcomes and newborn outcomes	Delay >3 h increased severe maternal outcomes. Family decision-making, financial barriers, and provider-related delays were major contributors.
Hopkins et al. [24]	United States	Retrospective cohort	281 term singleton inductions starting with misoprostol	Misoprostol followed by a Foley catheter	Misoprostol alone	Time to delivery, cesarean delivery, NICU admission, maternal and neonatal morbidity	Sequential Foley use after misoprostol was associated with longer labour, higher cesarean delivery, and higher NICU admission.
Bączek et al. [25]	Poland	Retrospective case-control study	4,350 women included in the oxytocin-versus-no-oxytocin analysis, selected from a source database of 29,455 term deliveries	Oxytocin augmentation	No oxytocin augmentation	Factors associated with oxytocin use, delivery outcomes, and neonatal outcomes	Oxytocin augmentation was associated with higher BMI, preinduction, epidural anaesthesia, longer labour, more operative deliveries, and more low Apgar scores.
Patel et al. [26]	Multi-country Global Network sites	Prospective registry cohort	150,848 singleton live births	Presence of all 8 care-around-delivery indicators	Incomplete care-around-delivery indicators	Early neonatal mortality	Presence of all 8 indicators was associated with reduced early neonatal mortality, although some individual indicators showed confounding by neonatal risk status.
Desplanches et al. [27]	France	Retrospective population-based propensity-score analysis	7,589 nulliparous women with operative vaginal delivery	Mediolateral episiotomy	No mediolateral episiotomy	OASIs, neonatal condition at birth, NICU admission	Mediolateral episiotomy was associated with lower OASIs and improved infant condition at birth in forceps/spatula delivery.
Verhaeghe et al. [28]	France	Randomised controlled trial	236 women with ultrasound-confirmed occiput posterior fetal position	Manual rotation at full dilation	Expectant management	Spontaneous vaginal delivery, operative delivery, cesarean delivery, morbidity	Manual rotation did not increase spontaneous vaginal delivery or reduce operative or cesarean delivery.

Risk of Bias Assessment

Risk of bias was assessed using design-specific tools. The nine non-randomised studies were evaluated using ROBINS-I across seven domains: confounding, participant selection, intervention classification, deviations from intended interventions, missing data, outcome measurement, and selection of reported results [18]. The randomised controlled trial was assessed separately using RoB 2 across five domains: randomisation, deviations from intended interventions, missing outcome data, outcome measurement, and selection of the reported result [17]. Overall risk ranged from moderate to serious among the non-randomised studies, mainly due to confounding by indication, baseline obstetric risk, emergency status, and provider or facility factors. The randomised trial was judged to have some concerns because of its open-label design. Table 3 shows the domain-level ROBINS-I judgements for the nine non-randomised studies. 

**Table 3:** ROBINS-I assessment of non-randomised studies ROBINS-I: Risk of Bias in Non-randomised Studies of Interventions.

Study	Confounding	Participant selection	Intervention classification	Deviations from intended interventions	Missing data	Outcome measurement	Selection of reported result	Overall risk
Zheng et al. [19]	Moderate	Low	Low	Low	Low	Low	Low	Moderate
Kebede et al. [20]	Moderate	Moderate	Low	Moderate	Moderate	Moderate	Low	Moderate
Sertel et al. [21]	Serious	Moderate	Low	Moderate	Moderate	Moderate	Low	Serious
Birendra and Jigyasa [22]	Serious	Serious	Low	Moderate	Serious	Moderate	Moderate	Serious
Kiruja et al. [23]	Moderate	Moderate	Low	Moderate	Moderate	Moderate	Low	Moderate
Hopkins et al. [24]	Serious	Moderate	Low	Moderate	Low	Low	Low	Serious
Bączek et al. [25]	Serious	Moderate	Low	Moderate	Moderate	Moderate	Low	Serious
Patel et al. [26]	Moderate	Low	Low	Moderate	Moderate	Moderate	Low	Moderate
Desplanches et al. [27]	Moderate	Moderate	Low	Low	Low	Low	Low	Moderate

Table 4 shows the RoB 2 assessment of the randomised controlled trial, which was judged to have some concerns overall, mainly because of its open-label design.

**Table 4:** RoB 2 assessment of the randomised controlled trial RoB 2: Risk of Bias 2.

Study	Randomisation process	Deviations from intended interventions	Missing outcome data	Outcome measurement	Selection of reported result	Overall risk
Verhaeghe et al. [28]	Low risk	Some concerns	Low risk	Some concerns	Low risk	Some concerns

Labour Induction, Augmentation, and Fetal Monitoring

The included studies reported varied effects of intrapartum interventions on maternal and neonatal outcomes [19,20,24,25]. Vaginal misoprostol combined with Foley balloon insertion was associated with a shorter induction-to-delivery interval and a higher proportion of vaginal deliveries within 24 hours, whereas sequential Foley catheter use after misoprostol was associated with longer labour, a higher rate of caesarean delivery, and increased NICU admission [19,24]. Oxytocin augmentation was associated with longer labour, more operative deliveries, and a higher frequency of low Apgar scores in the included database study [25]. Continuous electronic fetal monitoring in low-risk labour was associated with higher caesarean and instrumental delivery rates without a clear improvement in immediate neonatal outcomes [20]. These findings were interpreted within the specific populations, comparators, and clinical settings of the individual studies and support the selective, indication-based use of intrapartum interventions.

Planned Delivery, Emergency Care, and Operative Procedures

Two retrospective studies evaluated planned and emergency delivery in pregnancies complicated by placenta previa or placenta accreta spectrum, with sample sizes of 128 and 32 women, respectively [21,22]. In both studies, planned delivery was associated with more favourable neonatal and surgical outcomes than emergency delivery [21,22]. A prospective cohort study reported that a caesarean delay exceeding three hours was associated with increased severe maternal outcomes and identified family decision-making, financial barriers, and provider-related factors as contributors to the delay [23]. Mediolateral episiotomy was associated with lower obstetric anal sphincter injury rates during operative vaginal delivery, particularly forceps or spatula delivery [27]. Manual rotation of the occiput posterior position did not increase spontaneous vaginal delivery or reduce operative or caesarean delivery [28]. Each intervention was represented by a single primary study, except planned versus emergency delivery, which was represented by two retrospective studies.

Discussion

The findings from this systematic review were synthesised from 10 completed studies that investigated labour management, interventions during labour, emergency obstetric care, operative delivery, and neonatal outcomes. The results show that obstetric interventions can be beneficial for certain outcomes when they are used appropriately and as clinically indicated, but when used routinely or delayed, they may increase the risk to the mother and/or her neonate. The included evidence found inconsistent results for interventions such as induction methods, oxytocin augmentation, fetal monitoring, timing of caesarean section, and operative vaginal procedures. This justifies the need to consider individualised decision-making about intervention use in all labouring patients instead of uniform intervention use [29,30]. The studies of labour induction and augmentation indicated a strong association with the clinical context. Pharmacological plus mechanical induction did not seem to prolong the induction process, whereas sequential induction using a Foley catheter after misoprostol was associated with prolonged labour and an increased incidence of caesarean section [19,24]. This may be because women who need more ripening after misoprostol may also be a harder-to-induce cohort. Likewise, some positive delivery outcomes, such as shorter labour and better Apgar scores, were linked to oxytocin augmentation, while some negative outcomes, such as longer labour and increased operative delivery, were linked to oxytocin augmentation in a few instances [29,31]. The results of this study indicate that augmentation should be based on labour progress, maternal condition, and fetal status.

The evidence also demonstrated the significance of patient selection when using fetal monitoring. In low-risk labour, continuous electronic fetal monitoring was linked to an increase in caesarean and instrumental deliveries, but there was no improvement in the immediate outcomes of the newborn [20,29]. This could be attributed to misinterpretation of fetal heart rate abnormalities, resulting in unindicated operative delivery. Intermittent auscultation is still acceptable in low-risk women when clinical factors permit, and continuous monitoring might be more fitting in high-risk labour or when fetal compromise is suspected [30]. This is significant in environments that lack resources for surgery or neonatal care or have limited staffing. The studies on placenta previa, placenta accreta spectrum, and caesarean delay demonstrated that planned care is especially crucial in high-risk obstetric situations. Planned delivery was associated with better neonatal and surgical outcomes in placenta previa, particularly placenta accreta spectrum [22]. The rates of prematurity, NICU admission, invasive ventilation, and hysterectomy were higher in emergency cases [21]. Likewise, caesarean delivery more than three hours after the onset of labour was linked to adverse maternal outcomes, particularly when family decision-making, financial constraints, or provider-related factors delayed delivery [32,33]. The results highlight the importance of early diagnosis, referral planning, preparation of blood products, and rapid surgical decision-making in minimising preventable morbidity.

Other findings regarding operative vaginal delivery were also clinically significant. A decrease in obstetric anal sphincter injury during operative vaginal delivery and better infant condition at birth in forceps or spatula deliveries were linked to mediolateral episiotomy [27]. This indicates that episiotomy might have a protective function in selected high-risk operative deliveries but should not be performed routinely in all vaginal deliveries [34,35]. Manual rotation of the occiput posterior fetal position, however, failed to improve spontaneous vaginal delivery or decrease operative delivery [28]. The result indicates that not all biologically plausible procedures have measurable clinical benefits when tested in randomised studies [35]. The timing of delivery and the quality of care provided before birth affected neonatal outcomes in the studies. In the large registry-based study, the percentage of early neonatal mortality was associated with early initiation of breastfeeding, skin-to-skin care, clean delivery practices, and complete care-around-delivery indicators [26,30]. However, a few results revealed some surprising relationships with poorer outcomes, presumably because ill babies were more likely to have received extra care or to have been bathed later [26]. This underscores the need for careful interpretation of observational findings and the need to consider clinical severity as a potential confounder.

Limitations and Future Recommendations

This review has several limitations that should be considered when interpreting the findings. Most included studies were observational, retrospective, registry-based, or database-based, increasing the potential for confounding by indication, selection bias, and residual confounding. Women with greater baseline clinical severity were more likely to undergo emergency delivery, delayed caesarean delivery, sequential induction, operative vaginal delivery, or intensive neonatal care, making it difficult to distinguish the effect of the intervention from that of the underlying condition. The findings regarding planned versus emergency delivery were based on two small retrospective studies involving 128 and 32 women and may have been particularly affected by confounding by indication, as women requiring emergency delivery were likely to have had more severe clinical presentations. These associations should not be interpreted as evidence that planned delivery independently caused better maternal or neonatal outcomes. Retrospective studies also depended on the accuracy and completeness of medical records. The included studies differed in design, setting, population characteristics, intervention definitions, comparator groups, and maternal and neonatal outcome measures. Each intervention category was represented by only one or two primary studies, limiting the strength and generalisability of the conclusions. This heterogeneity precluded valid cross-study comparisons and pooled statistical analysis; therefore, a narrative synthesis was considered the most appropriate approach.

Future research should prioritise large, multicentre, prospective studies using standardised definitions of maternal morbidity, neonatal morbidity, operative delivery, severe maternal outcomes, NICU admission, Apgar score, and early neonatal mortality. Stronger adjustment for baseline risk is needed, especially in comparisons between planned and emergency care, delayed and timely caesarean delivery, and routine and selective intrapartum interventions. Where feasible and ethically acceptable, pragmatic randomised controlled trials should evaluate induction methods, fetal monitoring strategies, augmentation protocols, episiotomy use, and manual rotation in clearly defined risk groups. Future studies should also include long-term maternal and neonatal outcomes, cost-effectiveness analyses, patient-centred outcomes, implementation barriers, and health-system factors such as referral delays, surgical readiness, staffing, blood product availability, and neonatal support. Such evidence would help guide safe, timely, individualised, and resource-sensitive obstetric care.

## Conclusions

The findings of this systematic review indicate that maternal and neonatal outcomes associated with obstetric interventions vary according to clinical context, timing, patient risk, and the manner in which each intervention is applied. Evidence on combined induction strategies remains limited, with different outcomes reported across concurrent and sequential approaches. Continuous fetal monitoring, oxytocin augmentation, caesarean timing, operative vaginal procedures, and immediate newborn care practices also showed intervention-specific associations rather than uniform effects across settings. Planned delivery in placenta previa and placenta accreta spectrum was associated with more favourable outcomes than emergency delivery, although these findings were based on small observational studies and may have been influenced by underlying clinical severity. Mediolateral episiotomy was associated with a lower risk of obstetric anal sphincter injury in selected operative deliveries, whereas manual rotation did not alter the mode of delivery in the included trial. Each intervention category was represented by only one or two primary studies; the conclusions should therefore be interpreted cautiously and within the populations, comparators, and study designs evaluated. Further well-designed prospective and randomised studies are required before broad practice-level recommendations can be made.
